# Application of the Global Diet Quality Score in Chinese Adults to Evaluate the Double Burden of Nutrient Inadequacy and Metabolic Syndrome

**DOI:** 10.1093/jn/nxab162

**Published:** 2021-10-23

**Authors:** Yuna He, Yuehui Fang, Sabri Bromage, Teresa T Fung, Shilpa N Bhupathiraju, Carolina Batis, Megan Deitchler, Wafaie Fawzi, Meir J Stampfer, Frank B Hu, Walter C Willett, Yanping Li

**Affiliations:** National Institute for Nutrition and Health, Chinese Center for Disease Control and Prevention, Beijing, China; National Institute for Nutrition and Health, Chinese Center for Disease Control and Prevention, Beijing, China; Department of Nutrition, Harvard T.H. Chan School of Public Health, Boston, MA, USA; Department of Nutrition, Harvard T.H. Chan School of Public Health, Boston, MA, USA; Department of Nutrition, Simmons University, Boston, MA, USA; Department of Nutrition, Harvard T.H. Chan School of Public Health, Boston, MA, USA; Channing Division of Network Medicine, Department of Medicine, Brigham and Women's Hospital, Harvard Medical School, Boston, MA, USA; CONACYT—Health and Nutrition Research Center, National Institute of Public Health, Cuernavaca, Mexico; Intake – Center for Dietary Assessment, FHI Solutions, Washington, DC, USA; Department of Nutrition, Harvard T.H. Chan School of Public Health, Boston, MA, USA; Department of Global Health and Population, Harvard T.H. Chan School of Public Health, Boston, MA, USA; Department of Epidemiology, Harvard T.H. Chan School of Public Health, Boston, MA, USA; Department of Nutrition, Harvard T.H. Chan School of Public Health, Boston, MA, USA; Channing Division of Network Medicine, Department of Medicine, Brigham and Women's Hospital, Harvard Medical School, Boston, MA, USA; Department of Epidemiology, Harvard T.H. Chan School of Public Health, Boston, MA, USA; Department of Nutrition, Harvard T.H. Chan School of Public Health, Boston, MA, USA; Channing Division of Network Medicine, Department of Medicine, Brigham and Women's Hospital, Harvard Medical School, Boston, MA, USA; Department of Epidemiology, Harvard T.H. Chan School of Public Health, Boston, MA, USA; Department of Nutrition, Harvard T.H. Chan School of Public Health, Boston, MA, USA; Channing Division of Network Medicine, Department of Medicine, Brigham and Women's Hospital, Harvard Medical School, Boston, MA, USA; Department of Epidemiology, Harvard T.H. Chan School of Public Health, Boston, MA, USA; Department of Nutrition, Harvard T.H. Chan School of Public Health, Boston, MA, USA

**Keywords:** GDQS, double burden, China, metabolic syndrome, nutrient inadequacy

## Abstract

**Background:**

The double burdens of under- and overnutrition are changing the health of individuals and the economic and disease burdens in China. Poor diet plays an important role; however, a valid and easily operationalized metric that could capture the full range of characteristics of the diet that are relevant to both under- and overnutrition is lacking in China.

**Objectives:**

We aimed to examine the application of the Global Diet Quality Score (GDQS) to evaluate nutrient inadequacy and metabolic syndrome in different demographic groups of Chinese adults.

**Methods:**

A total of 35,146 individuals (men 14,978, women 20,168) aged >18 y from the 2010–2012 China National Nutrition and Health Survey were included. We scored the GDQS using average intakes of 25 food groups from 3 d of 24-h dietary recalls. Double burden was defined as coexisting metabolic syndrome and nutrient inadequacy.

**Results:**

Diet quality assessed by GDQS was significantly higher in urban than in rural residents (20.8 compared with 18.7), and increased with both educational level and household income (*P*-trends < 0.0001). A higher GDQS score was inversely associated with metabolic syndrome and nutrient inadequacy, or both (*P*-trends < 0.0001): multivariate adjusted ORs comparing extreme quintiles of GDQS were 0.79 (95% CI: 0.69, 0.91) for metabolic syndrome, 0.17 (95% CI: 0.14, 0.20) for nutrient inadequacy, and 0.59 (95% CI: 0.50, 0.69) for the double burden. These associations were consistent across different household income levels (*P*-interaction = 0.26), suggestively stronger in younger (<50 y), females, urban residents, and the more highly educated (*P*-interaction < 0.05) compared with their counterparts.

**Conclusions:**

A higher GDQS was inversely associated with a double burden of nutrient inadequacy and metabolic syndrome across various subgroups of Chinese adults. The finding supports the use of the GDQS in different demographic groups of Chinese adults to assess diet quality and nutritional status.

Rapid economic development, health promotion, and disease prevention strategies in China have led to better control of infectious diseases and marked reduction of undernutrition (1, 2). However, the overall burden of undernutrition and micronutrient deficiencies remains high in China. As reported by the China National Nutrition and Health Report 2015 (1), malnutrition affects 6% of adults and 9% of children and adolescents; the overall prevalence of anemia at age ≥6 y is 9.7%, and is 17.2% in pregnant women. In the same time period, rapid urbanization and industrialization in China have also led to a steep decline in physical activity and a dramatic shift in diet from traditional to Western dietary patterns (3, 4), which has been accompanied by marked increases in serum cholesterol concentrations (5), obesity (6), hypertension (7), and type 2 diabetes (8) as well as cardiovascular diseases and a variety of cancers.

To date, we lack a simple, easy-to-apply metric that could capture the full range of characteristics of the diet that are relevant to both under- and overnutrition in China. To address this problem, we developed the Global Diet Quality Score (GDQS) (9): a new diet quality assessment method to capture both under- and overnutrition. In brief, the GDQS is a food-based metric that incorporates a more comprehensive list of food groups than most existing diet metrics, and uses a simple method for scoring consumed amounts (9). The GDQS performed comparably with the Minimum Dietary Diversity–Women (MDD-W) (10) score in association with anthropometric and biochemical indicators of nutrient inadequacy; and comparably or better than the Alternative Healthy Eating Index (AHEI) (11) in capturing outcomes related to noncommunicable diseases (9). Different from the simple Dietary Diversity Score (12) and MDD-W, the GDQS classifies the foods into healthy and unhealthy categories with different direction of scoring based on epidemiological evidence of their health benefits/effects. Initially, the GDQS was aimed for use in nonpregnant, nonlactating women of reproductive age.

The aim of the current study was to examine the association between the GDQS and the double burden of malnutrition (both under- and overnutrition) in different demographic groups of Chinese adults. As defined by the WHO (13), the double burden of malnutrition is characterized by the coexistence of undernutrition with overweight and obesity, or diet-related noncommunicable diseases. For this analysis, we used overall nutrient inadequacy as an indicator of undernutrition, and metabolic syndrome as an indicator of overnutrition.

## Methods

### Study population

The 2010–2012 China National Nutrition and Health Survey (CNNHS) is a nationally representative cross-sectional study conducted by the Chinese Center for Disease Control and Prevention to assess the health and nutrition of Chinese civilians in 2010–2012 (14). The CNNHS 2010–2012 was conducted in 31 provinces, autonomous regions, and municipalities directly under the central government, throughout China (except Taiwan, Hong Kong, and Macao). Subjects were selected using a multistage, stratified cluster, random sampling method. A detailed description of the survey design is provided in previous publications (14).

In brief, the CNNHS 2010–2012 (14) selected 150 study sites from 4 strata (**Supplemental Figure 1**): 34 big cities, 41 medium and small cities, 45 general rural areas, and 30 poor rural areas applying a multistage, stratified cluster, random sampling method according to economic and social development characteristics as reported by the 2010 China Population Census. CNNHS 2010–2012 selected 6 communities/villages from each study site, and 75 households from each community/village. The data collections of the 2010–2012 CNNHS included questionnaires, clinical physical examination, and laboratory tests. All family members from the selected households were invited for blood draw sample collection. Of 120,226 invited participants aged ≥18 y, 104,098 provided complete data on physical examination, blood lipids, and fasting glucose tests. Thirty households within each set of 75 households were randomly selected to record their dietary intakes, which resulted in 35,146 participants for the current study. Household and family information was collected by face-to-face interviewer-administered questionnaires in the household, by trained investigators.

### Data collection and definition of nutrition inadequacy

Dietary information was collected using 24-h dietary recalls of all foods and beverages consumed, with the exception of oils and condiments, which were weighed. Collection of dietary data was carried out over 3 consecutive days (including 2 weekdays and 1 weekend day). For each dietary recall day, investigators went to the participant's home and helped to record food intake during the last 24 h. Pictures of typical portion size of usually consumed foods were shown to the participants to help them recall the food weight consumed. For mixed dishes, investigators asked the participants to recall the individual food ingredients that participants consumed. Investigators also weighed the household cooking oil and condiments (such as salt, soy sauce, or ketchup that were used to add flavor to food) at the beginning and end of the 3 d of recall for the 24-h dietary survey. Nutrient intakes were calculated using the China Food Composition Tables (FCT-2002/2004) (15, 16).

Probability of adequacy (PA) (17) of fiber, protein, and micronutrients (iron, thiamin, riboflavin, calcium, vitamin A, zinc, and vitamin C) was computed by applying the Chinese estimated average requirement cut-point (18–21). Mean PA, as a summary variable for nutrient adequacy, was formed by averaging all 9 PA values. Nutrient inadequacy was defined by mean PA <50%.

### GDQS scoring

The average food intakes of 3 d from dietary recalls were grouped into 25 food groups according to the GDQS coding method as described in detail by Bromage et al. (9). In brief, the GDQS consists of 16 healthy food groups (dark green leafy vegetables, cruciferous vegetables, deep orange vegetables, other vegetables, deep orange fruits, deep orange tubers, citrus fruits, other fruits, legumes, nuts and seeds, poultry and game meat, fish, whole grains, liquid oils, low fat dairy, and eggs), and 9 unhealthy food groups (white roots and tubers, processed meats, refined grains and baked goods, sugar-sweetened beverages, sweets and ice cream, juices, purchased deep fried foods, high fat dairy, and red meat). Quantity of intake for each food group is classified into 3 or 4 ranges. For healthy food groups, points between 0 to 4 are given to each level of intake depending on the food group. For unhealthy food groups, 2, 1, or 0 points are given for the intake levels, so that lower intake receives more points. The GDQS is a sum of all 25 GDQS food group scores, with a possible score range of 0 to 49 points. A higher score represents a healthier diet.

### Data collection and measurement of metabolic syndrome

Fasting body weight, height, and blood pressure were measured by trained investigators. Participants were requested to present to selected study sites during morning hours for anthropometric measurements. Height without shoes and weight in the fasting state were measured to an accuracy of 1 mm and 0.1 kg, respectively. Waist circumference was measured to the nearest 0.1 cm at the midpoint between the bottom of the rib cage and the top of the iliac crest at the end of exhalation using the skin touch measuring tape custom-made for CNNHS.

Subjects’ seated blood pressure was measured 3 times, using a mercury sphygmomanometer on the right arm after 5 min of rest, to the nearest 2 mmHg. The mean of the 3 measures was used for analysis. The cuff size was selected on the basis of the upper arm circumference.

Participants were invited for blood collection after ∼10–14 h overnight fast. The samples were centrifuged at 433 × *g* for 10 min at room temperature after being left standing for 30–60 min. Plasma glucose was measured using a spectrophotometer with glucose oxidase kit in the laboratory of local units of the Center for Disease Control and Prevention within 4 h of blood draw. Reagents were purchased from Beijing Zhongsheng Reagents Company. All local laboratories participated in the quality control program and passed the quality control testing of the National Center for Clinical Laboratories.

Serum was obtained from centrifuged blood samples and transported to the central laboratory of the National Institute for Nutrition and Health. Total cholesterol, HDL cholesterol, and triglycerides were measured by a Hitachi automatic biochemical analyzer (Model 7600) with reagents from Wako Pure Chemical Industries, Ltd. Reagents of the same batch were used and purchased from Beijing Zhongsheng Reagents Company. Serum cholesterol was measured using the cholesterol oxidase aminoantipyrin phenol method (CHOD-PAP); serum triglyceride was measured using the glycerol phosphate oxidase 4-chloric acid method.

According to the joint interim statement of the International Diabetes Federation Task Force on Epidemiology and Prevention; National Heart, Lung, and Blood Institute; American Heart Association; World Heart Federation; International Atherosclerosis Society; and International Association for the Study of Obesity (22), participants having ≥3 of the following conditions were defined as having metabolic syndrome: abdominal obesity (waist circumference ≥90 cm in men and ≥80 cm in women); high triglycerides (≥1.7 mmol/L); low HDL cholesterol (<1.04 mmol/L in men and <1.29 mmol/L in women); elevated blood pressure (systolic blood pressure ≥130 mmHg or diastolic blood pressure ≥85 mmHg or use of antihypertensive drugs in a patient with a history of hypertension); or hyperglycemia (fasting plasma glucose ≥5.6 mmol/L or physician diagnosis of diabetes).

### Statistical analysis

The 2010–2012 CNNHS was designed to provide accurate estimates of nutritional status in the Chinese population according to sex, age, and level of economic development. Applying the poststratification population sampling weights derived for the dietary surveys from the sampling probability of the 2010 Chinese population aged ≥20 y (based on census data), we estimated nationally representative population levels for intakes of foods and GDQS score, the prevalence of the outcomes as well as the main association analysis, except the stratified and interaction analyses, in considering the sampling weight was not representative of all subgroups.

Participants were divided into quintile categories of GDQS score. We used logistic regression models to estimate ORs and 95% CIs comparing participants in a given quintile category of GDQS with those in the lowest quintile. To quantify a linear trend, we conducted a Wald test for linear trend by assigning the median intake within each quintile and modeling this as a continuous variable. Multivariable models were adjusted for age, sex, income, education level, living area, job categories, smoking status, and energy intake.

Separate analyses were conducted for different specific subgroups, including sex, age, urban/rural, education, and income levels. The interaction between the GDQS score and each stratification variable was evaluated by adding a multiplicative factor in the logistic regression model and tested for significance using the −2log likelihood method.

All statistical analyses were conducted using SAS software, including the survey procedures software, version 9.4 (SAS Institute).

## Results

A total of 35,146 participants from CNNHS 2010–2012 aged ≥18 y, including 14,978 males and 20,168 females, were included in our study. The sample distribution according to age, living areas, educational level, income, and smoking status are shown in **Table 1**.

**TABLE 1:** Sample size according to the basic characteristics of study population (*n* = 35,146)

	Male	Female	Total
Total	14,978	20,168	35,146
Age, *n* (%)			
18–49 y	6085 (40.6)	9421 (46.7)	15,506 (44.1)
≥50 y	8893 (59.4)	10,747 (53.3)	19,640 (55.9)
Living area, *n* (%)			
Urban	7565 (50.5)	10,423 (51.7)	17,988 (51.2)
Rural	7413 (49.5)	9745 (48.3)	17,158 (48.8)
Educational level, *n* (%)			
Illiteracy	1001 (6.7)	3304 (16.4)	4305 (12.2)
Elementary	4067 (27.2)	6279 (31.1)	10,346 (29.4)
Middle school	5805 (38.8)	6433 (31.9)	12,238 (34.8)
High school	2674 (17.9)	2815 (14.0)	5489 (15.6)
Community college and above	1431 (9.6)	1337 (6.6)	2768 (7.9)
Annual per capita household income, *n* (%)			
<10,000 yuan	7338 (49.0)	9786 (48.5)	17,124 (48.7)
10,000–14,999 yuan	2733 (18.2)	3782 (18.8)	6515 (18.5)
15,000–24,999 yuan	2670 (17.8)	3531 (17.5)	6201 (17.6)
≥25,000 yuan	1558 (10.4)	2084 (10.3)	3642 (10.4)
No answer	679 (4.5)	985 (4.9)	1664 (4.7)
Smoking, *n* (%)			
Yes	9259 (61.8)	932 (4.6)	10,191 (29.0)
No	5719 (38.2)	19,236 (95.4)	24,955 (71.0)

**Table 2** presents the average quantity of consumption for each of the 25 GDQS food groups. For the following food groups, the majority of the study population fell into the lowest category for quantity of consumption for each food group: legumes (63.9%), nuts and seeds (86.1%), whole grain (92.5%), fish (66%), deep orange fruits (99.3%), citrus fruits (90.2%), other fruits (75.7%), low fat dairy (99.9%), poultry (72.1%), cruciferous vegetables (75.6%), deep orange vegetables (80.4%), deep orange tubers (93.5%), high fat dairy (88.8%), juice (98.2%), processed meat (91.6%), white roots and tubers (61.2%), sugar-sweetened beverages (99.4%), sweets and ice cream (92.1%), and purchased deep fried foods (87.6%). Whereas for the following food groups the majority of the sample occupied the highest category for quantity of consumption: liquid oils (87.3%), dark green leafy vegetables (47.1%), other vegetables (66%), red meat (47.3%), and refined grains and baked goods (99.98%).

**TABLE 2:** Quantity of intake for each food group and the distribution of the GDQS subscore for each food group included in the GDQS in the study population (*n* = 35,146)^1^

	Average intake (mean (SE),^2^ g/d	Scoring ranges^3^ (cutoffs, g/d) low/middle/high	GDQS subscores	Low %	Middle %	High %
Legumes	12.3 (0.2)	<10/10–39/>39	0, 2, 4	63.90	27.74	8.36
Nuts and seeds	3.8 (0.2)	<4/4–16/>16	0, 2, 4	86.11	5.41	8.48
Whole grains	3.6 (0.2)	<4/4–17/>17	0, 1, 2	92.49	1.80	5.71
Liquid oils	36.8 (0.5)	<2/2–8/>8	0, 1, 2	8.04	4.66	87.29
Dark green leafy vegetables	62.9 (0.7)	<10/10–39/>39	0, 2, 4	35.25	17.62	47.13
Fish and seafood	22.7 (0.4)	<16/16–64/>64	0, 1, 2	65.98	20.80	13.23
Deep orange fruits	0.5 (0.0)	<28/28–114/>114	0, 1, 2	99.29	0.65	0.06
Citrus fruits	7.0 (0.3)	<18/18–75/>75	0, 1, 2	90.23	6.85	2.92
Other fruits	30.1 (0.8)	<26/26–106/>106	0, 1, 2	75.66	14.68	9.66
Low fat dairy	0.4 (0.2)	<34/34–140/>140	0, 1, 2	99.92	0.02	0.06
Poultry and game meat	15.5 (0.3)	<12/12–48/>48	0, 1, 2	72.09	15.70	12.21
Eggs	22.3 (0.3)	<7/7–29/>29	0, 1, 2	42.57	25.01	32.41
Cruciferous vegetables	14.5 (0.3)	<11/11–44/>44	0, 0.25, 0.5	75.57	11.96	12.47
Deep orange vegetables	12.1 (0.3)	<11/11–44/>44	0, 0.25, 0.5	80.39	9.83	9.78
Deep orange tubers	4.2 (0.2)	<14/14–57/>57	0, 0.25, 0.5	93.46	3.79	2.75
Other vegetables	154.9 (1.1)	<21/21–84/>84	0, 0.25, 0.5	8.54	25.45	66.01
High fat dairy	14.1 (0.4)	<34/34–140/140–734/>734	0, 1, 2, 0	88.75	7.94	3.29/0.02
Red meat	59.3 (0.6)	<12/12–48/>48	0, 1, 0	26.56	26.16	47.29
Juice	2.0 (0.1)	<35/35–141/>141	2, 1, 0	98.21	1.41	0.38
Processed meat	3.0 (0.1)	<8/8–31/>31	2, 1, 0	91.58	4.54	3.88
White roots and tubers	32.7 (0.5)	<25/25–101/>101	2, 1, 0	61.09	30.32	8.59
Refined grains and baked goods	294.1 (1.1)	<4/4–14/>14	2, 1, 0	0.01	0.00	99.98
Sugar-sweetened beverages	0.9 (0.2)	<52/52–210/>210	2, 1, 0	99.40	0.52	0.07
Sweets and ice cream	3.3 (0.1)	<11/11–46/>46	2, 1, 0	92.06	5.64	2.30
Purchased deep fried foods	7.9 (0.3)	<10/10–40/>40	2, 1, 0	87.64	4.45	7.92

1 GDQS, Global Diet Quality Score.

2 Average intake and proportion were weighted by sampling weight.

3 Scoring ranges: the 3 categories here are the low, middle, and high separated by a solidus; for high fat dairy, 4 categories were classified: low, lower middle, high middle, and high (from left to right).

**FIGURE 1:**
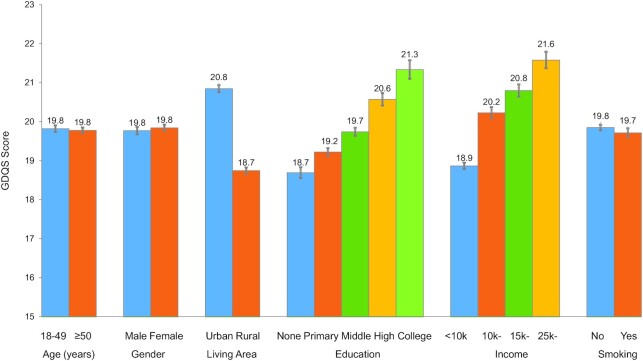
Average GDQS score according to the basic characteristics of study population (*n* = 35,146) (sample size of each subgroup is listed in Table 1). GDQS, Global Diet Quality Score.

**FIGURE 2:**
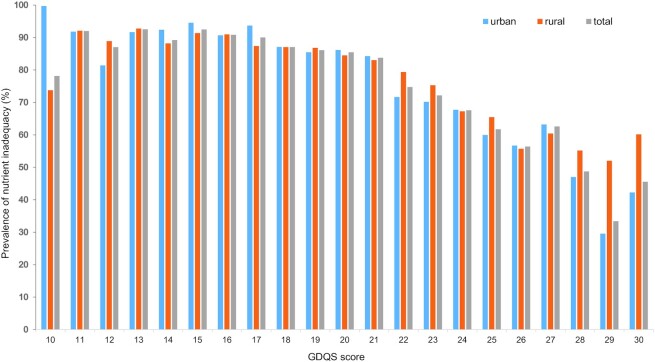
Weighted prevalence of nutrient inadequacy according to GDQS score of study population (*n* = 35,146). GDQS, Global Diet Quality Score.

**FIGURE 3:**
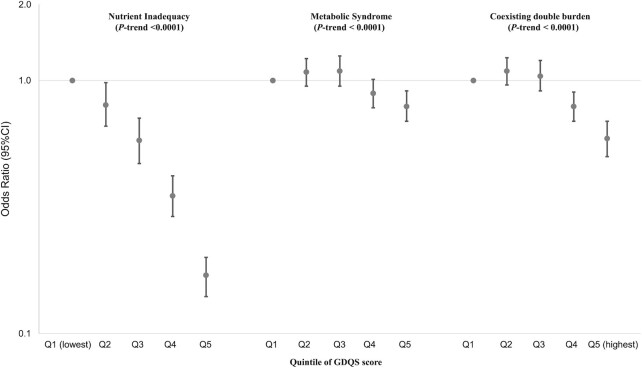
ORs (95% CI) of nutrient inadequacy and metabolic syndrome according to GDQS score in study population (*n* = 35,146). GDQS, Global Diet Quality Score.

The overall average GDQS score was 19.8 (SE = 0.03), similar for males and females (**Figure 1**). Urban residents had a significantly higher GDQS score (20.8 for urban compared with 18.7 for rural; *P* < 0.0001). The average GDQS score significantly increased with educational and family income levels (Figure 1). The overall prevalence of nutrient inadequacy based on the 9 nutrients was 81.7%, which gradually decreased with an increasing GDQS score (**Figure 2**). Nutrient inadequacy presented in 90.9% of population with the lowest quintile of GDQS and 61.6% in the highest quintile (**Table 3**). Compared with those in the lowest quintile for GDQS, the OR of nutrient inadequacy was 0.17 (95% CI: 0.14, 0.20) for individuals in the highest quintile (**Figure 3**); per SD increasing GDQS was associated with a 52% lower odds of nutrient inadequacy (OR: 0.48; 95% CI: 0.45, 0.51) (Table 3). People within the highest quintile of GDQS also had a 21% lower odds of metabolic syndrome compared with the lowest GDQS (OR: 0.79; 95% CI: 0.69, 0.91) (Figure 3). A total of 22.0% of the study population had both nutrient inadequacy and metabolic syndrome (coexisting in the same individual), which was similar across the lowest 3 GDQS quintile groups (22.3–25.2%; Table 3), but this proportion was significantly lower in the 2 highest GDQS quintiles. The OR of the double burden of nutrient inadequacy and metabolic syndrome, comparing the extreme quintiles, was 0.59 (95% CI: 0.50, 0.69; Figure 3).

**TABLE 3:** ORs (95% CI) of nutrient inadequacy and metabolic syndrome according to GDQS score in the study population (*n* = 35,146)^1^

Quintile of GDQS score	Q1 (lowest quintile)	Q2	Q3	Q4	Q5 (highest quintile)	*P*-trend	Per SD
*n* (sample)	7080	7726	6510	6955	6875		
GDQS score (median)	14.5	17.5	19.75	22.0	25.25		
Nutrient inadequacy							
Prevalence	90.9	89.0	85.8	79.8	61.6		
Age- and sex- adjusted OR	1.0 (Ref.)	0.81 (0.68, 0.96)	0.61 (0.50, 0.73)	0.39 (0.33, 0.47)	0.16 (0.14, 0.19)	<0.0001	0.47 (0.44, 0.50)
Multivariate adjusted OR	1.0 (Ref.)	0.82 (0.68, 0.98)	0.62 (0.51, 0.75)	0.40 (0.33, 0.48)	0.17 (0.14, 0.20)	<0.0001	0.48 (0.45, 0.51)
Further adjusted energy intake	1.0 (Ref.)	0.80 (0.66, 0.98)	0.58 (0.47, 0.71)	0.35 (0.29, 0.42)	0.17 (0.14, 0.20)	<0.0001	0.48 (0.45, 0.51)
Metabolic syndrome							
Prevalence	24.9	27.9	29.0	25.6	24.6		
Age- and sex- adjusted OR	1.0 (Ref.)	1.15 (1.02, 1.30)	1.21 (1.06, 1.39)	1.03 (0.91, 1.17)	0.98 (0.86, 1.12)	0.29	0.98 (0.94, 1.02)
Multivariate adjusted OR	1.0 (Ref.)	1.08 (0.95, 1.22)	1.09 (0.95, 1.25)	0.89 (0.78, 1.02)	0.79 (0.69, 0.91)	<0.0001	0.90 (0.86, 0.95)
Further adjusted energy intake	1.0 (Ref.)	1.08 (0.95, 1.22)	1.09 (0.95, 1.25)	0.89 (0.78, 1.01)	0.79 (0.69, 0.91)	<0.0001	0.90 (0.86, 0.95)
Coexisting double burden							
Prevalence	22.3	25.2	25.1	20.5	16.4		
Age- and sex- adjusted OR	1.0 (Ref.)	1.15 (1.02, 1.31)	1.14 (1.00, 1.31)	0.89 (0.78, 1.01)	0.67 (0.58, 0.78)	<0.0001	0.85 (0.82, 0.89)
Multivariate adjusted OR	1.0 (Ref.)	1.08 (0.95, 1.22)	1.03 (0.90, 1.18)	0.77 (0.67, 0.88)	0.55 (0.47, 0.64)	<0.0001	0.79 (0.75, 0.83)
Further adjusted energy intake	1.0 (Ref.)	1.09 (0.96, 1.23)	1.04 (0.91, 1.20)	0.79 (0.69, 0.90)	0.59 (0.50, 0.69)	<0.0001	0.81 (0.77, 0.85)

1 Multivariate adjusted model adjusted for age, sex, income, educational level, living area (urban compared with rural), and smoking status. GDQS, Global Diet Quality Score; Q, quintile.

The associations between GDQS and the double burden of nutrient inadequacy and metabolic syndrome were consistent across different household income levels (*P*-interaction = 0.26; **Table 4**) but were significantly stronger in younger than older individuals (*P*-interaction = 0.02), in females than males (*P*-interaction < 0.001), and in urban than rural residents (*P*-interaction < 0.001) (Table 4). Comparing the extreme quintiles of the GDQS, the OR for the double burden of nutrient inadequacy and metabolic syndrome was 0.71 (95% CI: 0.61, 0.81) in those with low education levels; 0.48 (95% CI: 0.43, 0.55) for middle education levels; and 0.39 (95% CI: 0.27, 0.58) for high education levels (Table 4).

**TABLE 4:** ORs (95% CI) of the double burden of nutrient inadequacy and metabolic syndrome according to GDQS score stratified by basic characteristics of the study population (*n* = 35,146)^1^

Quintile of GDQS score	Q1 (lowest)	Q2	Q3	Q4	Q5 (highest)	*P*-trend	*P*-interaction
Age							
18–49 y	1.0 (Ref.)	1.03 (0.91, 1.16)	0.95 (0.83, 1.08)	0.74 (0.65, 0.85)	0.49 (0.42, 0.57)	<0.0001	0.02
≥50 y	1.0 (Ref.)	0.96 (0.88, 1.06)	0.99 (0.89, 1.09)	0.86 (0.78, 0.96)	0.58 (0.52, 0.65)	<0.0001	
Gender							
Male	1.0 (Ref.)	1.08 (0.96, 1.22)	1.06 (0.94, 1.21)	0.90 (0.79, 1.02)	0.61 (0.53, 0.70)	<0.0001	<0.001
Female	1.0 (Ref.)	0.92 (0.84, 1.02)	0.91 (0.82, 1.01)	0.77 (0.69, 0.86)	0.52 (0.46, 0.59)	<0.0001	
Living area							
Urban	1.0 (Ref.)	0.89 (0.78, 1.01)	0.87 (0.77, 0.99)	0.71 (0.63, 0.80)	0.48 (0.42, 0.55)	<0.0001	<0.001
Rural	1.0 (Ref.)	1.00 (0.91, 1.10)	0.99 (0.89, 1.10)	0.88 (0.78, 0.98)	0.65 (0.56, 0.75)	<0.0001	
Annual per capita household income							
<10,000 yuan	1.0 (Ref.)	1.00 (0.91, 1.10)	0.95 (0.86, 1.06)	0.84 (0.75, 0.94)	0.55 (0.48, 0.64)	<0.0001	0.26
10,000–14,999 yuan	1.0 (Ref.)	1.00 (0.83, 1.21)	1.09 (0.90, 1.32)	0.78 (0.64, 0.94)	0.60 (0.49, 0.73)	<0.0001	
15,000–24,999 yuan	1.0 (Ref.)	0.95 (0.76, 1.18)	0.93 (0.75, 1.15)	0.75 (0.61, 0.93)	0.54 (0.43, 0.66)	<0.0001	
≥25,000 yuan	1.0 (Ref.)	0.82 (0.59, 1.12)	0.83 (0.60, 1.13)	0.84 (0.63, 1.13)	0.49 (0.36, 0.65)	<0.0001	
Educational level^2^							
Low	1.0 (Ref.)	0.95 (0.85, 1.06)	0.92 (0.83, 1.04)	0.88 (0.78, 0.99)	0.71 (0.61, 0.81)	<0.0001	<0.001
Middle	1.0 (Ref.)	1.01 (0.90, 1.13)	0.98 (0.87, 1.10)	0.77 (0.68, 0.86)	0.48 (0.43, 0.55)	<0.0001	
High	1.0 (Ref.)	0.77 (0.51, 1.15)	0.89 (0.60, 1.32)	0.58 (0.39, 0.85)	0.39 (0.27, 0.58)	<0.0001	

1 Multivariate adjusted model adjusted for age, sex, income, educational level, living area (urban compared with rural), smoking status, and energy intakes except the stratified variables. GDQS, Global Diet Quality Score; Q, quintile.

2 Education level: Low = illiteracy or elementary; Middle = middle school; High = high school, community college, and above.

## Discussion

Based on these large-scale, nationally representative dietary and health data of Chinese adults, we observed inverse associations between a simple, easy-to-use diet quality score designed for global use and odds of the double burden of nutrient inadequacy and metabolic syndrome in different demographic groups of Chinese adults. Our findings support the application of the GDQS score, originally developed for use in nonpregnant, nonlactating women of reproductive age, as a valid method to capture the double burden of under- and overnutrition of women and men across different age, income, and educational groups in China.

The concurrent double burden of undernutrition and overnutrition has been reported in many developing countries, especially in countries experiencing rapid economic and nutrition transitions (4); whereas undernutrition often remains a major problem in poor and less developed rural areas, increases in sedentary behaviors and Westernized dietary practices can lead to obesity in the cities. Despite this geographical contrast, the double burden of undernutrition and overnutrition can also coexist within the same family and even within the same individuals; this coexistence poses further challenges for interventions. In Egypt (23), undernutrition and stunting are common in Upper Egypt, the poorest region in Egypt, whereas the prevalence of overweight is highest in urban Lower Egypt. At the same time, children living in the urban area of Upper Egypt had a significantly higher likelihood of being stunted and overweight, illustrating the coexistence of the double burden of undernutrition and overnutrition within the same child living in a poor area characterized by rapid urbanization. In Brazil and Russia (24), dual-burden households (households that include both underweight and overweight individuals in the same household) were more likely to be urban and low income than households with only overweight members, because rapid urbanization is linked to poor diet and reduced physical activity (25). Approximately half of the overweight children younger than 5 y, and 1 of every 4 overweight children aged 5–19 y living in poor rural areas of China coexist with stunting (26). Limited dietary diversity and intake of high-energy-dense foods were notably observed in stunted overweight children (27). Nearly 40% of Chinese adults aged 18–59 y experienced overweight/obesity and micronutrient deficiency simultaneously (28). Studies in adults in Burkina Faso (West Africa) (29, 30) indicated that a “traditional” dietary pattern, characterized by a higher intake of local cereals, legumes, and traditional green leafy vegetables, was a significant contributing factor to the double burden of malnutrition, where nutritional deficiency indicators included underweight, iron depletion, and vitamin A deficiency, and overnutrition indicators included obesity, hypertension, hyperglycemia, and dyslipidemia (29). In our study, we observed 27.7% with concurrent nutrient inadequacy and metabolic syndrome, which was associated with poor diet quality, as reflected by the GDQS. Preventing both under- and overnutrition simultaneously in the same population is a remarkable public health challenge, because providing adequate food to prevent undernutrition can in turn enable an obesogenic environment. The findings highlight the importance of nutritional quality in future dietary interventions.

Previous dietary scores that have been developed mainly focus either on prevention of chronic diseases or on identifying risk of malnutrition with limited food groups. To capture dietary characteristics that would associate with both nutrient adequacy and prevention of chronic diseases, the GDQS was constructed to consider 16 healthy and 9 unhealthy food groups (9). The validation studies in multiple countries in women of reproductive age indicated that GDQS was a measurement well suited to capture metabolic risk, comparable to the AHEI-2010 (11); GDQS also compared well with the MDD-W (10) in its ability to measure diet quality, and to capture nutrient inadequacy (9). The lack of need to use a food composition table for analysis of the data collected is another merit of GDQS that should be highlighted; which is important for its broad application worldwide.

The major limitation of our study is the cross-sectional design, which does not permit a sequence of temporality to be established for dietary GDQS and the double burden of nutrient inadequacy and the metabolic syndrome, which precludes the potential for reverse causality to be eliminated. Future prospective cohort studies are warranted to verify our findings. Another limitation is that the 3 d of dietary records could not catch the potential variations of food intakes in different seasons, which would influence the metric performance more in individuals from subsistence-oriented households, such as rural residents and less-educated populations. The present study is the largest study of diet and the double burden of under- and overnutrition in China. Strengths of the current study include the highly trained study staff to ensure the standardization of data collection. National representativeness, including 31 provinces of China, is another strength.

In conclusion, higher diet quality, as evaluated with GDQS, was associated with a lower likelihood of both nutrient inadequacy and metabolic syndrome, with stronger associations in younger, male, urban residents and those with a higher educational level. These findings support the use of GDQS to guide application of dietary strategies for the improvement of the double burden of under- and overnutrition burdens in China.

## Supplementary Material

nxab162_Supplemental_File

## Notes

Funding for the research was provided by FHI Solutions, recipient of a Bill & Melinda Gates Foundation grant to support Intake – Center for Dietary Assessment. The 2010– 2012 China National Nutrition and Health Survey was supported by the Special Fund for Health-Scientific Research in the Public Interest (No. 20120212) from the National Health and Family Planning Commission of the People’s Republic of China.

Author disclosures: YL has received research support from the California Walnut Commission and Swiss Re Management Ltd. TTF is an associate editor of the Journal of Nutrition and played no role in the Journal’s evaluation of the manuscript. All other authors report no conflicts of interest.

Published in a supplement to the Journal of Nutrition. Publication costs for this supplement were funded by the Bill & Melinda Gates Foundation in conjunction with FHI Solutions, recipient of a Bill & Melinda Gates Foundation grant to support Intake – Center for Dietary Assessment. The opinions expressed in this publication are those of the authors and are not attributable to the sponsors or the publisher, Editor, or Editorial Board of *The Journal of Nutrition*. The Supplement Coordinators for the supplement publication were Megan Deitchler, Intake – Center for Dietary Assessment at FHI Solutions, Washington, DC; and Sabri Bromage, Harvard T.H. Chan School of Public Health, Boston, MA.

Supplemental Figure 1 is available from the “Supplementary data” link in the online posting of the article and from the same link in the online table of contents at https://academic.oup.com/jn.

The GDQS research initiative was launched by Intake – Center for Dietary Assessment. The research was led by Harvard T.H. Chan School of Public Health, Department of Nutrition and carried out in collaboration with researchers at the National Public Health Institute (INSP), Mexico. Funding for the research was provided by FHI Solutions, recipient of a Bill & Melinda Gates Foundation grant to support Intake – Center for Dietary Assessment.

Abbreviations used: AHEI, Alternative Healthy Eating Index; CNNHS, China National Nutrition and Health Survey; GDQS, Global Diet Quality Score; MDD-W, Minimum Dietary Diversity–Women; PA, probability of adequacy.
