# Supplementary material for: Application of the Global Diet Quality Score in Chinese Adults to Evaluate the Double Burden of Nutrient Inadequacy and Metabolic Syndrome
Source: J Nutr. 2021 Oct 23;151(Suppl 2):93S–100S. doi: 10.1093/jn/nxab162 (PMC8542094; doi:10.1093/jn/nxab162)
Supplement: nxab162_Supplemental_File [file nxab162_supplemental_file.docx]

“Application of the Global Diet Quality Score in Chinese population to evaluate the double burden of nutrient inadequacy and metabolic syndrome” He Y et al. Online Supplemental Material.

**Supplemental Figure 1: Map of study sites in the 2010-2012 CNNHS**

**
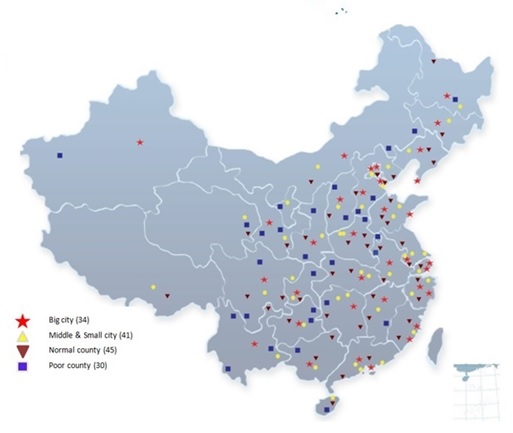
**
